# X-linked myotubular myopathy in Rottweiler dogs is caused by a missense mutation in Exon 11 of the *MTM1* gene

**DOI:** 10.1186/s13395-014-0025-3

**Published:** 2015-01-27

**Authors:** G Diane Shelton, Branden E Rider, Georgina Child, Sophia Tzannes, Ling T Guo, Behzad Moghadaszadeh, Emily C Troiano, Bianca Haase, Claire M Wade, Alan H Beggs

**Affiliations:** Department of Pathology, School of Medicine, University of California San Diego, La Jolla, CA USA; Division of Genetics and Program in Genomics, The Manton Center for Orphan Disease Research, Boston Children’s Hospital and Harvard Medical School, 300 Longwood Ave., Boston, MA 02115 USA; Small Animal Specialist Hospital, North Ryde, NSW Australia; Faculty of Veterinary Science, The University of Sydney, Sydney, NSW 2006 Australia

**Keywords:** Congenital myopathy, Myotubularin, Canine myopathy, Animal model

## Abstract

**Background:**

Congenital and inherited myopathies in dogs are faithful models of human muscle diseases and are being recognized with increasing frequency. In fact, canine models of dystrophin deficient muscular dystrophy and X-linked myotubular myopathy are of tremendous value in the translation of new and promising therapies for the treatment of these diseases. We have recently identified a family of Australian Rottweilers in which male puppies were clinically affected with severe muscle weakness and atrophy that resulted in early euthanasia or death. X-linked myotubular myopathy was suspected based on the early and severe clinical presentation and histopathological changes within muscle biopsies. The aim of this study was to determine the genetic basis for myopathy in these dogs and compare and contrast the clinical presentation, histopathology, ultrastructure, and mutation in this family of Rottweiler dogs with the previously described myotubular myopathy in Labrador retrievers.

**Results:**

Histopathology, histochemistry, and ultrastructural examination of muscle biopsies from affected Rottweiler puppies were consistent with an X-linked myotubular myopathy. An unusual finding that differed from the previously reported Labradors and similar human cases was the presence of excessive autophagy and prominent autophagic vacuoles. Molecular investigations confirmed a missense mutation in exon 11 of *MTM1* that was predicted to result in a non-functional phosphatase activity*.* Although the clinical presentations and histopathology were similar, the *MTM1* p.(Q384P) mutation is different from the p.(N155K) mutation in exon 7 affecting Labrador retrievers with X-linked myotubular myopathy.

**Conclusions:**

Here we describe a second pathogenic mutation in *MTM1* causing X-linked myotubular myopathy in dogs. Our findings suggest a variety of *MTM1* mutations in dogs as seen in human patients. The number of *MTM1* mutations resulting in similar severe and progressive clinical myopathy and histopathological changes are likely to increase as canine myopathies are further characterized.

## Background

The centronuclear myopathies (CNMs) are a group of pathologically defined disorders that characteristically have a high proportion of small myofibers with centrally placed nuclei [1-3]. Classical centronuclear myopathies in humans have been associated with dominant mutations in the large GTPase *DNM2* gene [4] while recessive cases may be due to mutations of amphiphysin 2, encoded by *BIN1* [5], and of the ryanodine receptor (RyR1) gene, *RYR1* [6]. An important, well-defined sub-group of CNMs is X-linked myotubular myopathy (XLMTM) associated with mutations in the *MTM1* gene [7]. The protein product, myotubularin, is a ubiquitously expressed 603 amino acid phosphoinositide phosphatase that is essential in a wide variety of cellular signaling pathways governing diverse cellular functions including apoptosis and vesicle trafficking [8]. In skeletal muscle, myotubularin is localized at the terminal cisternae of the sarcoplasmic reticulum (SR) where its function is critical for regulating the lipid composition of membranes at the triads [9]. Abnormal membrane tubulation in myotubularin-deficient muscle leads to altered T-tubule and triad morphology and defective excitation contraction coupling, which is thought to play a major role in the initial pathophysiological events leading to weakness [10,11]. Clinical onset of XLMTM is at or near birth with males born with severe generalized hypotonia and weakness with respiratory insufficiency [12]. Affected infants are typically areflexic and hypotonic with little or no anti-gravity movements. The condition is not dystrophic as myofibers remain largely intact and serum creatine kinase (CK) activities are within the reference range or only mildly elevated.

In veterinary medicine, the first mutation associated with classical CNM was identified in young Labrador retrievers. A widely distributed exonic SINE insertion in the *PTPLA* gene segregated with autosomal recessive CNM in these dogs [13]. Mutations in the *PTPLA* gene have not been reported to date in human cases of CNM, however, mutation in the *BIN1* exon 11 acceptor splice site was recently shown to be the cause of a rapidly progressive and fatal centronuclear myopathy in both a consanguineous human family and in young Great Dane dogs with a progressive CNM [14]. While specific mutations have not yet been identified, CNM has also been reported in a family of young Manchester Terriers (G.D. Shelton, unpublished observations), in a young Border Collie [15], and in an Arabian foal [16].

In 2008, a form of CNM resembling XLMTM was described in a family of Labrador Retrievers from Canada [17]. Molecular investigation confirmed that these dogs indeed did have an *MTM1* mutation and represented a faithful model of human XLMTM [18]. Male puppies from two kindreds were presented for evaluation of progressive weakness and muscle atrophy beginning in the first few months of life. Pathological changes in muscle biopsies were consistent with a myotubular myopathy and a missense mutation, p.(N155K), was identified in exon 7 of canine *MTM1.* Here we describe, compare, and contrast the clinical presentation, histopathology, muscle ultrastructure, and a second independent mutation of *MTM1* in a family of Rottweiler dogs from Australia.

## Methods

### Animals

**Figure 1:**
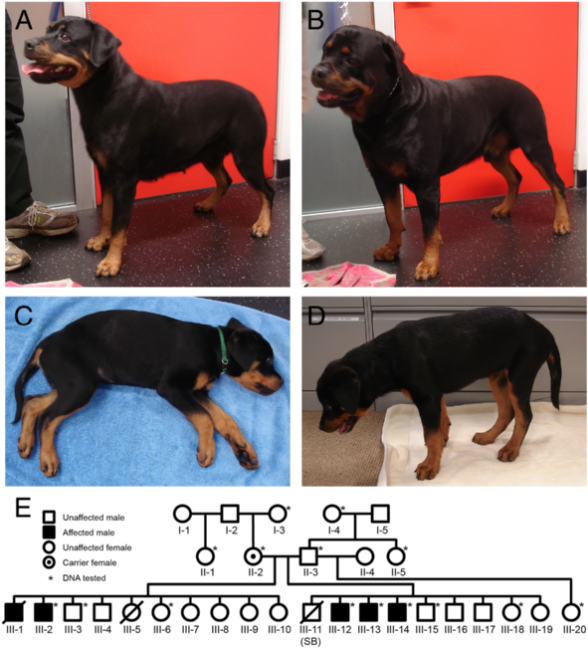
**Clinical presentations and family history of affected dogs.** Photographs of the healthy dam II-2 **(A)**, sire II-3 **(B)**, and two affected male littermates III-12 **(C)** and III-13 **(D)** at 12 weeks of age. **(E)** Pedigree of the two affected Rottweiler litters with male puppies that presented with progressive muscle weakness. The 15 members whose DNA was available for analysis are indicated by an asterisk. Squares = males, circles = females, SB = still born. Filled symbols indicate clinically affected. The dot in the center of II-2 indicates her genetically confirmed status as a carrier for the *MTM1* mutation illustrated below.

All dogs reported in this communication were privately owned pets receiving clinical veterinary care and no experimental procedures were carried out on live animals. All research on tissue biopsies and blood samples was performed on specimens collected and submitted for clinical diagnostic purposes and the owners and breeders were informed and gave consent to study tissues for the welfare of the breed. Four male puppies, aged 8 to 13 weeks, were presented to the Small Animal Specialist Hospital, North Ryde, Australia (GC and ST) for evaluation of progressive muscle weakness. The affected puppies were from two different litters with the same dam and sire (Figure 1). The sire of both litters had sired two other litters from different dams with no reported abnormalities in the offspring. DNA for genetic testing was collected from all four affected puppies and 10 unaffected first-degree relatives. Additional unaffected control samples were obtained from The Rottweiler Blood Bank, The University of Manchester, Manchester, UK, The Faculty of Veterinary Science, University of Sydney, Australia, and the Animal Specialist Hospital, North Ryde, Australia. None of the dogs in the unaffected control groups showed clinical signs of a neuromuscular disease.

### Histopathology, histochemistry, and immunofluorescence staining

Immediately after collection, muscle biopsy specimens were either wrapped in a saline-dampened gauze sponge and immediately refrigerated or immersion-fixed in 10% neutral buffered formalin. All specimens were shipped by a courier service under refrigeration to the Comparative Neuromuscular Laboratory, University of California San Diego. Immediately on receipt, unfixed specimens were flash-frozen in isopentane precooled in liquid nitrogen and stored at -80°C until further processing. Control muscles were all histologically normal specimens from the frozen tissue archive of the Shelton laboratory. Cryosections (8 μm) were cut and processed by a standard panel of histochemical stains and reactions [19]. Mouse monoclonal antibodies against slow (1:10, NCL-MHCs, Novocastra, Newcastle, England) and fast (1:10, NCL-MHCf, Novocastra) myosin heavy chains were used for fiber typing. Peroxidase substrate kits (DAB, Vector SK-4100, Vector Laboratories, Burlingame, CA, USA) and Vector red substrate kits (Vector, SK-5100) were used for color development according to package instructions.

Additional cryosections (8 μm) were cut and processed by indirect immunofluorescence. Sections were fixed in chilled acetone for 5 min, then incubated with a rabbit polyclonal antibody against the T-tubule marker, dihydropyridine receptor (DHPR), DHPRα1 (ab58552, 1:100 dilution, Abcam, Cambridge, UK) and a mouse monoclonal antibody against the SR marker, RyR1 (ab2827, 1:100 dilution; Abcam, Cambridge, UK). Rabbit polyclonal antibodies against LC3 N terminal (1:100, Abgent AP1802a, Abgent, San Diego, CA, USA) and ubiquitin (1:250, Dako 2015-02, Dako Denmark A/S, Glostrup, Denmark) were used as markers for autophagy. Details of further processing are as previously described [18].

### Electron microscopy and quantification of triad structures

Following collection, muscles were immersed in ice cold 5% glutaraldehyde for 4 h, cut into 2 to 3 mm^3^ blocks, and fixed for an additional 2 h before post-fixation in 1% osmium tetroxide for 1 h. The samples were dehydrated in increasing ethanol concentrations and finally propylene oxide, and then embedded in araldite resin. Thick sections (1 μm) were prepared and examined by light microscopy after staining with toluidine blue-basic fuchsin. Ultra-thin sections (60 to 65 nm) were cut and collected on 100 or 200 square mesh grids, stained with uranyl acetate and lead citrate, and examined with a Zeiss 10 electron microscope. For quantification of triad structures in a representative affected dog and an age-matched control dog, longitudinal sections were evaluated at 9,900 magnification. A triad structure was defined as the typical T-tubule density and at least one adjacent cistern. The first 10 fields encountered with no artifact were photographed and the number of triads per field counted.

### DNA extraction and *MTM1* gene sequencing and analysis

Genomic DNA was isolated from muscle biopsies and EDTA anticoagulated peripheral blood by standard procedures. The 15 transcribed exons, plus 81 to 278 bp of flanking intronic sequences, of the canine *MTM1* gene were polymerase chain reaction (PCR) amplified and Sanger sequenced using primer set 2 as described [18]. The canine *MTM1* c.1151A > C variant was screened for in relatives of the proband and unaffected control populations using a Custom TaqMan® SNP Genotyping Assay (Life Technologies custom assay AH89RBZ, Thermo Fisher Scientific, Waltham, MA USA) on an ABI 7300 real time PCR platform (Thermo Fisher Scientific). Canine DNA and protein sequence variants are specified relative to reference sequences XM_850116.3 and XP_855209.1 where cDNA numbering is based on designating the first ‘A’ of the initiating methionine as position 1.

### Variant interpretation and mutational modeling

Potential pathogenic consequences of the *MTM1* c.1151A > C variant were assessed using SIFT (http://sift.jcvi.org/) [20], PolyPhen 2 v2.2.2 (http://genetics.bwh.harvard.edu/pph2/) [21], and SuSPect (http://www.sbg.bio.ic.ac.uk/suspect/) [22] and effects on protein structure were modeled using Phyre2 (http://www.sbg.bio.ic.ac.uk/phyre2/) [23] and visualized using Jmol, an open-source JAVA viewer for chemical structures in 3D (http://www.jmol.org/). Amino acid alignments were performed using the ClustalW algorithm in MacVector version 13.04 (MacVector Inc., Cary NC, USA), using the following reference sequences for comparison. For myotubularins: human (*Homo sapiens*), NP_000243.1; monkey (*Macaca mulatta*), NP_001248647; dog (*Canis familiaris*), XP_005641991; cow (*Bos taurus*), NP_001193354; chicken (*Gallus gallus*), XP_004940916; xenopus (*Xenopus tropicalis*), AAH94184.1; zebra fish (*Danio rerio*), NP_001032773.1; drosophila (*Drosophila melanogaster*), AAF52327.1. For myotubularin-related proteins: *MTM1*, NP_000243.1; *MTMR1*, NP_003819.1; *MTMR2*, Q13614.4; *MTMR3*, CAG30411.1; *MTMR4*, NP_004678.3; *MTMR5*, O95248.3; *MTMR6*, NP_004676.3; *MTMR7*, EAW63816.1; *MTMR8*, Q96EF0.1; *MTMR9*, Q96QG7.1; *MTMR10*, Q9NXD2.3; *MTMR11*, A4FU01.2; *MTMR12*, Q9C0I1.2; *MTMR13*, Q86WG5.1.

## Results

### Severe and progressive myopathy in related litters of Rottweiler puppies

A 13-week-old male Rottweiler (III-2 from Figure 1) from a litter of 10 puppies (6 females and 4 males) was referred for progressive weakness. Two pups from this first litter were previously lost; the first was a female that died within a few weeks of birth (unknown cause), and the second a male pup (III-1) euthanized by the attending veterinarian at 9 weeks of age for an initial alteration in facial expression and exercise intolerance progressing to an inability to bear weight. In the affected pup evaluated (III-2), clinical signs of weakness had been apparent since acquisition from the breeder at 8 weeks of age. Clinical examination at referral revealed normal mentation, generalized mild to moderate reduction in muscle mass without myalgia, and profound tetraparesis with an inability to support weight. Cervical ventroflexion occurred in sternal recumbency. Spinal pain was absent. Hyporeflexia and hypotonia were noted in all limbs, and were symmetrical in severity. Sensory abnormalities were not detected. There were no cranial nerve deficits. Conscious proprioception (proprioceptive paw positioning) was intact. CK was mildly elevated (2119 U/l, reference 0-400). Multiple muscle and peripheral nerve biopsies were obtained via open surgical biopsy under general anesthesia. After sample collection, the puppy was euthanized for humanitarian reasons and at the owners’ request due to the severe nature of the disease.

Subsequently, three male Rottweiler puppies (Figure 1E, puppies III-12, 13, and 14) were presented with a history of rapidly progressive generalized weakness and poor muscle development. The puppies were from a second litter of eight surviving puppies (6 male and 2 female, 1 additional male stillborn) produced after the second mating of the dam and sire of puppy III-2 (Figure 1E). Two of the affected puppies (III-12 and III-13) were presented for evaluation at 11 weeks of age. Both were regarded as normal with respect to their littermates in activity level and muscle development until 7 weeks of age when the owner noted both puppies showed difficulty in holding their heads up and tired easily. The puppies remained bright, alert, and normally responsive, were keen to play with their littermates but were increasingly unable to do so. Muscle development was poor compared to their littermates despite comparable size, a normal appetite, and food intake. Weakness was exacerbated by any stress and cold. The third puppy (III-14) was regarded as normal until 9 weeks of age and was reported to be less active at 11 weeks of age. This puppy presented 2 weeks after his littermates (at 13 weeks of age).

Clinical findings were similar for all three affected puppies. All puppies were bright, friendly, and normally responsive. Bent or crinkled vibrissae were identified at an early age, and not apparent in the normal littermates. Poor development or reduced muscle mass compared to normal littermates was noted in the limb muscles, paraspinal muscles, and masticatory muscles. The most severely affected puppy (Figure 1C, puppy III-12) was unable to stand for more than 10 s. All affected puppies had marked cervical weakness, were unable to lift their heads when standing (Figure 1D) and were unable to raise the head when sternally recumbent after any exertion. The puppies adopted a hunched thoracolumbar posture with a stiff limbed stance and narrow base when standing. A stiff, short-stride gait was evident before sitting or lying down. In a recumbent position the puppies would follow the activities of other puppies or people with their eyes rather than moving their head or neck. When ambulatory no evidence of ataxia or proprioceptive deficit was evident. Voluntary motor movement was present in all limbs and limb positioning appropriate, but all showed reduced muscle strength. When standing or encouraged to walk the respiratory rate of all the affected puppies would increase, they would pant and the heart rate would increase significantly (>200 bpm).

Postural reactions including proprioceptive paw positioning were normal if the puppies’ weight was supported. Patellar reflexes were absent in the two most severely affected puppies (Figure 1E, puppies III-12 and III-13) and reduced in the third affected puppy (III-14). Flexion reflexes were present in all four limbs in all puppies but weak in puppies III-12 and III-13. Cranial nerve examination was normal in all three puppies. CK was normal or very mildly increased (104, 348, 277 U/L; reference 0 to 200).

In view of the severe and rapidly progressive clinical signs, and diagnosis of myopathy in a related dog, all three puppies were euthanized at 11 weeks of age (III-12), 12 weeks of age (III-13), and 13 weeks of age (III-14), respectively. Skeletal and cardiac muscle and blood samples were collected at necropsy for histology, immunohistochemistry, and genetic analysis.

### Histopathology, histochemistry, and immunohistochemical findings suggest affected puppies have a form of centronuclear myopathy with excessive autophagy

**Figure 2:**
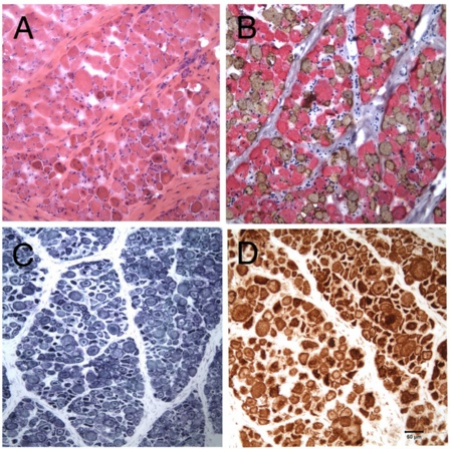
**Pathological findings in skeletal muscle from an affected puppy are suggestive of a diagnosis of XLMTM or other form of CNM.** Cryosections of the biceps femoris muscle from the affected puppy III-2 were evaluated with a standard panel of histochemical stains and reactions. Marked variability in myofiber size was noted **(A)** with numerous hypotrophic fibers containing central nuclei. Most hypotrophic fibers were of type 1 as determined by staining with antibodies against slow (brown stain, type 1 fibers) and fast (pink stain, type 2 fibers) myosin heavy chains **(B)**. Dense central and subsarcolemmal deposits were noted with the oxidative reactions SDH **(C)** and COX **(D)**.

Cryosections from the triceps, extensor carpi radialis, tensor fascia lata, cranial tibial, and biceps femoris muscles were evaluated from puppy III-2 (Figure 2). There was a marked variability in myofiber size in all muscles examined with fiber diameters in the range of 12 to 60 μm (Figure 2A). Normal canine biceps femoris contains 41% type 1, 58% type 2, and 1% type 2C fibers [24]. Biceps femoris from the affected dogs displayed type 1 fiber predominance (73% type 1 fibers and 27% type 2 fibers), which was evident by antibody staining for fast and slow myosin heavy chains (Figure 2B). Most type 1 fibers were hypotrophic with prominent central nuclei. The hypotrophic fibers contained central accumulations and subsarcolemmal rings, the so-called ‘necklace fibers’, that stained dark blue with the oxidative reactions nicotinamide adenine dinucleotide - tetrazolium reductase (not shown), succinate dehydrogenase (SDH, Figure 2C) and cytochrome C oxidase (COX, Figure 2D). Necrotic fibers were rare. No abnormalities were detected in the radial, femoral, and sciatic nerve sections, and gross and microscopic examination of the hearts from several affected dogs revealed no evidence of cardiomyopathy or other recognizable lesions. Based on these pathological changes, a congenital myopathy including centronuclear myopathy or myotubular myopathy was diagnosed. Cryosections were also evaluated from the affected male puppies III-12 and III-14 with similar morphologic findings.

**Figure 3:**
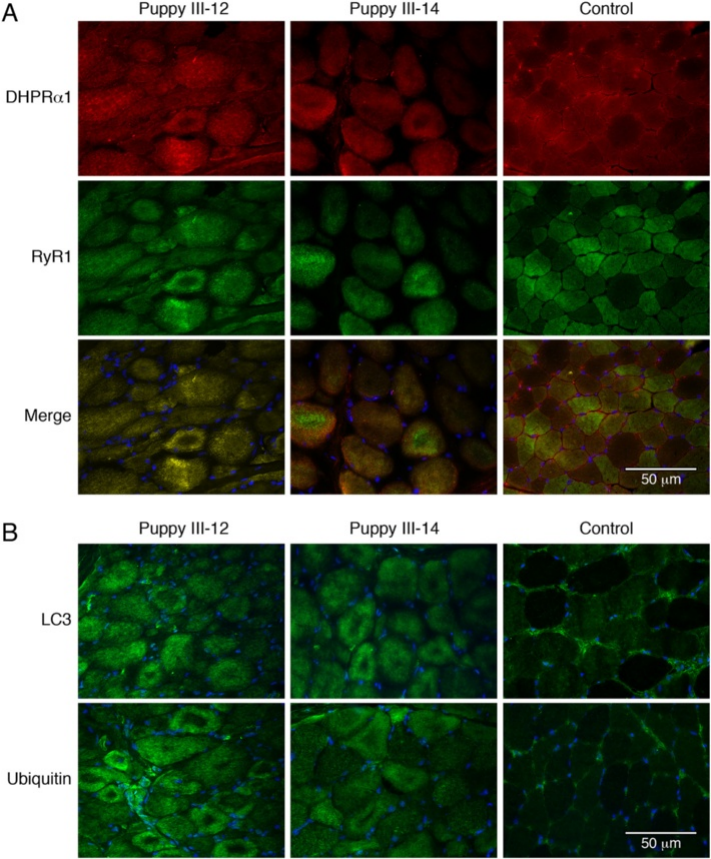
**Abnormal staining patterns for triad proteins and markers of autophagy in skeletal muscles from affected dogs. (A)** Immunofluorescence staining of cryosections from the biceps femoris muscles of Rottweiler puppies affected with XLMTM (puppies III-12 and III-14). Irregular staining patterns were noted in affected puppies using antibodies against DHPRα1 (T-tubule marker) and RyR1 (SR marker) compared to control muscle. **(B)** Compared to control muscle, dense and irregular staining patterns were also noted following staining with the autophagy markers LC3 and ubiquitin.

Immunofluorescence staining of muscle cryosections from affected puppies, using antibodies against markers for T-tubules (DHPRα1) and SR (RyR1), revealed an abnormal localization of both structures compared to control muscle (Figure 3). T-tubules and surrounding SR were concentrated in irregular densities within numerous myofibers, but not in the control muscle (Figure 3A). Antibodies against LC3 and ubiquitin, markers for autophagy, showed prominent dense staining in most myofibers from the affected puppies but not in control muscle (Figure 3B).

### Abnormal triads, autophagic vacuoles, and mitochondrial accumulations were found in affected muscles from Rotweillers with XLMTM

**Figure 4:**
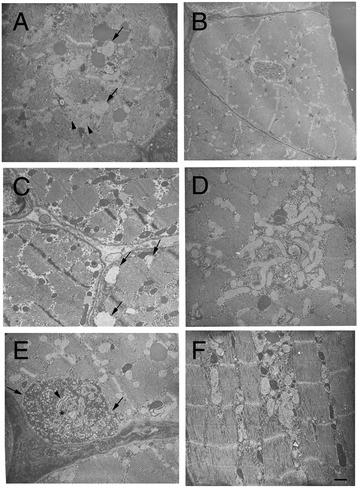
**Ultrastructural studies of the biceps femoris muscle from a Rottweiler puppy affected with XLMTM (puppy III-12).** Prominent features include myofibrillar disarray, SR profiles (arrows), autophagosomes (arrowheads) and other degenerating organelles **(A)**, centrally located nuclei **(B)**, dilated tubular structures (arrows, **C**), and mitochondrial accumulation with interspersed glycogen granules **(D)**. A cross-section of a double membrane bound (arrows) autophagic vacuole is shown with entrapment of organelles including T-tubule (arrowhead) and junctional SR (asterisk) profiles, other membranes of undetermined origin, mitochondria, and glycogen granules **(E)**. Multiple adjacent autophagic vacuoles occupying abnormal fiber regions are shown in **(F)**. Scale bar in lower right corner of F = 0.2 μm for **A**, **D** and **E**, 0.6 μm for **B**, 0.3 μm for **C**, and 0.3 μm for **F**.

Ultrastructural examinations (Figure 4) were performed on the biceps femoris muscles of puppies III-12 and III-14. Representative images in Figure 4 are from puppy III-12. Normal appearing triads were not observed in the sections from the affected puppies, while 18 to 20 triads per field were present in the control dog sections (not shown). Large areas of myofibrillar disarray were prominent containing degenerating organelles, and lysosomal and tubular structures (Figure 4A). Centrally located nuclei were confirmed (Figure 4B). Dilated tubular structures (T-tubules and SR profiles) were present throughout the sections (Figure 4C) and clusters of mitochondria with interspersed glycogen granules were found (Figure 4D). Remarkably, prominent abundant autophagic vacuoles (Figure 4E,F) containing heterogeneous contents including small dense bodies studded with glycogen particles, membrane fragments, myeloid structures, and debris were noted.

### Identification of a novel *MTM1* mutation in affected dogs

**Figure 5:**
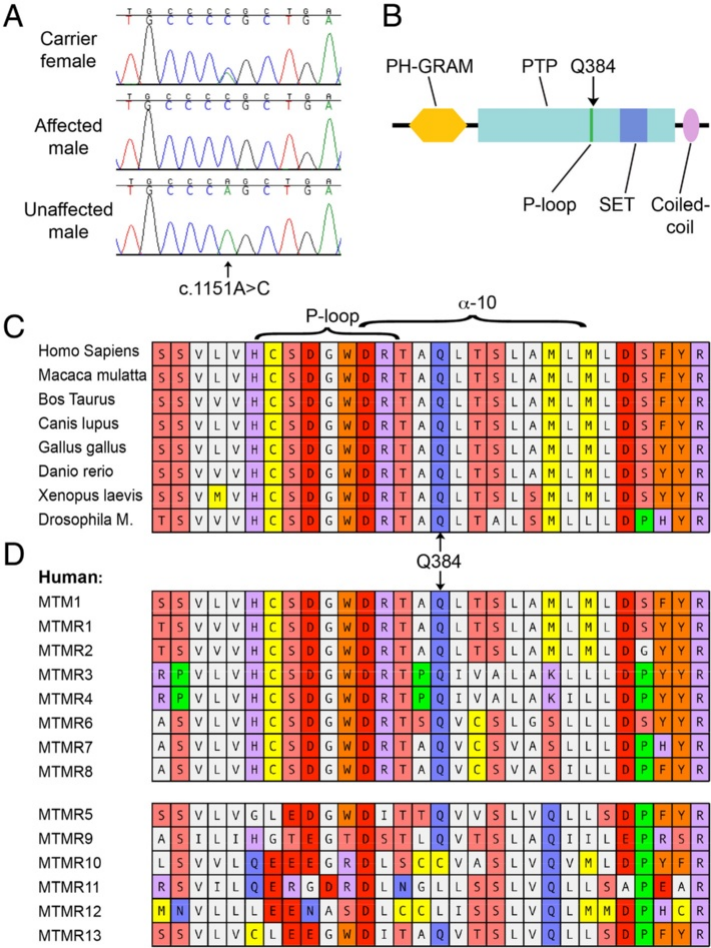
**Mutation analysis of the** ***MTM1*** **gene in affected and unaffected Rottweilers. (A)** DNA sequence tracings from Sanger sequencing of a portion of *MTM1* exon 11 centered on cDNA position 1151 illustrating mutation of adenine to a cytosine in a hemizygous affected puppy (middle), compared with an unaffected male littermate (bottom) and his carrier mother (top). **(B)** Schematic of full-length myotubularin with major domains and motifs showing the location of the wild type glutamine (Q) at position 384. **(C, D)** Alignments of a 30 amino acid stretch of myotubularins from different species **(C)**, and from each of the first 13 human myotubularin related proteins **(D)**, centered about the position of Q384. **(D)** The six enzymatically inactive myotubularin related proteins are separated by a space from the eight active forms. Residues constituting the phosphatase active site (P-loop) and α helix 10 are indicated by brackets above.

All 15 exons and between 81 and 278 bp of flanking intronic sequences of the canine *MTM1* gene were PCR amplified from the genomic DNA of one of the affected puppies and subjected to Sanger sequencing. The puppy’s *MTM1* coding sequence completely matched the published reference sequence XM_850116.3 with the exception of a single base substitution of C for A in exon 11 at position 1151 on the coding portion of the cDNA, designated *MTM1* c.1151A > C (Figure 5A). This variant is predicted to introduce a non-conservative missense mutation replacing a glutamine at position 384 with a proline, designated p.(Q384P). Further PCR amplification and sequencing of exon 11 in the DNAs from 13 additional first degree relatives of the affected puppy, and one unrelated unaffected control revealed that all four affected males were hemizygous for this variant and the dam of these two affected litters was a heterozygous carrier, while all the remaining dogs carried only the wild type A allele at this position.

To investigate the occurrence and potential distribution of the *MTM1* c.1151A > C variant among healthy Rottweilers, a Custom TaqMan® SNP Genotyping Assay was developed to distinguish the two alternate alleles. Application of this assay to the genomic DNAs of 57 Rottweilers (30 male and 27 female) previously tested for rabies titers through the United Kingdom’s Pet Travel Scheme [25], as well as 19 additional Rottweilers from Australia, including four females, 14 males, and one of uncertain gender, revealed that all of the 107+ X chromosomes represented by these cohorts carried only the wild type A allele.

To assess the likelihood that the non-conservative replacement of the acidic amino acid glutamine with the cyclic amino acid proline at position 384 causes a pathogenic alteration in myotubularin structure and function, we subjected this mutation to several predictive algorithms. Residue Q384 is highly conserved and invariant among all the known myotubularins in species ranging from mammals to birds, fish, amphibians, and even invertebrates such as drosophila (Figure 5C). Canine *MTM1* p.(Q384P) is predicted ‘damaging’ by SIFT (score of 0.00, with Median Sequence Conservation = 3.06 based on 146 unique sequences, where scores <0.05 are considered damaging), ‘probably damaging’ by PolyPhen-2 (score 1.000, where >0.85 is classified probably damaging), and a SuSPect score of 89, with disease-associated single amino acid variants predicted to have a score above 50 [22]. In fact, inspection of the SuSPect scores for all of the possible amino acid substitutions for residues 370-418 reveals a region of the protein encompassing the phosphatase active site with particularly high scores (mean 80.3 +/- 20) versus overall scores for the entire protein (mean 48.2 +/- 27), suggesting that this region of the protein is particularly sensitive to missense changes.

### Molecular modeling of the *MTM1* p.(Q384P) mutation

**Figure 6:**
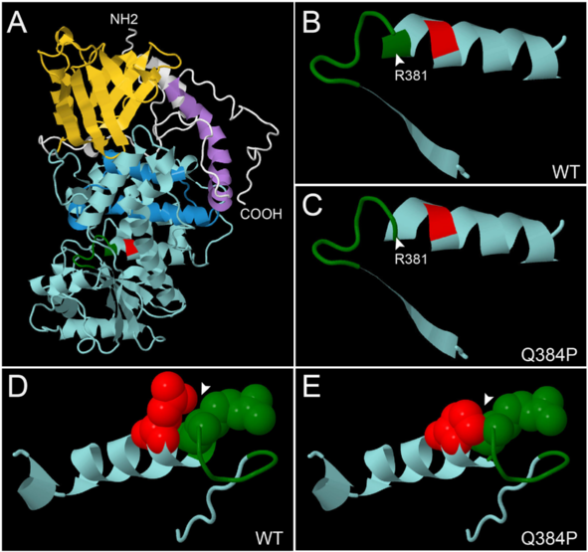
**Structural predictions of the effects of the** ***MTM1*** **p.(Q384P) mutation.** Jmol visualizations of predicted structural models of full-length canine myotubularin generated by Phyre2 **(A)** show the PH-GRAM domain (yellow) oriented at the top and the phosphatase domain (light blue) below. The SET and coiled coil domains are shown in blue and purple, respectively (as in Figure 5B). The P-loop is colored green and the position of Q384 is shown in red. The region encompassing β-sheet 14, the P-loop, and α-10 (residues 370-394), is shown in isolation for the wild type protein **(B)**, and for the p.(Q384P) mutation **(C)**. Note loss of alpha helical structure at arginine 381 (indicated). Repeating the Phyre2 structural predictions for just the 30 residue peptide including positions 369-398 and modeling space-filling atoms for positions 381 and 384 illustrates predicted steric interference between R381 and mutated P394 **(E)**, but not wild type Q384 **(D)** (arrowheads).

The human and canine myotubularin proteins are collinear with no gaps with 96% identity and 98% similarity. We used Phyre2 [23] to predict the protein structure of canine myotubularin by homology modeling. The server generated a robust structural model based on the crystal structure of MTMR2 using as its template RCSB Protein Data Bank entry 1ZSQ [26]. The positive alignment encompassed residues 33-543 of canine myotubularin with 100% confidence based on 69% identity in the aligned regions. Not surprisingly, given the high degree of evolutionary conservation, the resulting model predicts that canine myotubularin has essentially the same structure as human myotubularin, with a conserved P-loop at the enzyme’s phosphatase active site (residues 375-381) and a conserved alpha helix (α-10) immediately distal to it (Figure 6A) [26]. Residue Q384 is part of α-10, and is only three amino acids distal to the P-loop (Figures 5B,C, 6A). Examination of the region encompassing amino acids 370-394, including β-sheet 14, the P-loop, and α-10 predicts that the alpha helical structure of α-10 extends proximally to include the last residue of the P-loop at R381 (Figure 5B). Mutating Q384 to proline is predicted to disrupt this alpha helix, leading to R381 adopting a non-helical coil structure (Figure 6B, C). This is supported by clash analysis, which predicts that the change from glutamine to proline at position 384 leads to moderate interference with the side chain of R381 when just the 30 amino acids from position 369-398 are modeled (Figure 6D,E).

Given the structurally constrained nature of the P-loop, one might predict that this alteration should abolish, or at least modify, the enzymatic activity at this active site. Further support for this hypothesis comes from examination of the cognate Q384 position across the 13 most similar myotubularin-related proteins. The glutamate is conserved across all seven enzymatically active members of the family, but is replaced by a cysteine in MTMR10 and a lysine in MTMR11 and MTMR12, representing three of the six known enzymatically inactive myotubularins (Figure 5D).

## Discussion

Here we report a second genetically proven canine homolog of XLMTM due to mutation of the *MTM1* gene. Both the previously reported Labrador retriever mutation, *MTM1* p.(N155K), and this Rottweiler mutation, *MTM1* p.(Q384P), represent missense changes replacing one amino acid with another. p.(N155K) altered a residue in exon 7, located in the linker region between the PH-GRAM and the phosphatase domains and was associated with reduced levels of myotubularin protein. Unfortunately, due to issues of cross-reactivity (possibly with MTMR1 or MTMR2) with available antibodies, we were unable to reliably evaluate myotubularin localization or levels in the affected Rottweiler’s muscles, although western blotting suggested that myotubularin levels were similar in affected and wild type dogs and the staining pattern co-localized, as expected, in necklace fibers with that of the RyR1 (data not shown). Nevertheless, the p.(Q384P) mutation is predicted to dramatically alter, and possibly abolish, phosphatase activity so we posit that this leads to production of a stable, but catalytically inactive protein. This exact residue has not been reported mutated in human patients with XLMTM, however, missense mutations of S376 and G378 (both within the P-loop), and S387, A389, and L391 (all within the adjacent α-10 helix), have been reported. Of 13 patients in the *MTM1* Leiden Open Variation database [27] with missense mutation of one of these five residues, 10 were reported to have a severe phenotype similar to that seen in patients with *MTM1* null mutations, while three were of unknown severity.

Although a proven canine *MTM1* null mutation has not yet been reported, it is worth noting that both the Labrador retrievers and Rottweilers exhibited similar severe clinical phenotypes, with onset of a generalized, progressive, and fatal myopathy in the second to third month of life. Breeding of the Labrador *MTM1* p.(N155K) mutation onto a Beagle genetic background (that is, ‘Labbes’) has had no discernable impact on severity of the phenotype or natural history of the disease [28], suggesting that clinical expression of these mutations may be largely independent of breed.

Histopathology in the CNMs, including XLMTM, may be similar and include excessive variability in myofiber size with numerous hypotrophic fibers, central nuclei in myofibers reminiscent of myotubes, and the presence of ‘necklace fibers’. The necklace fibers, which are indicative of XLMTM, may appear as a ring of increased staining around the periphery of H&E stained myofibers, but are most apparent upon reaction with oxidative stains [29]. The necklaces are positive for SDH or COX (Figure 2C, D) and stain positively for components of the triad such as DHPR, and RyR1 (Figure 3A). These, and other abnormal staining intensities using antibodies against DHPR and RyR1, are typically found in XLMTM in the Labrador retrievers [18] and the Rottweilers presented with similar findings. However, a striking finding unique to the Rottweiler dogs was the presence of intensely staining myofibers with the autophagy markers LC3 and ubiquitin (Figure 3B) and this correlated with the presence of abundant autophagosomes and autophagic vacuoles upon ultrastructural examination (Figure 4E,F).

Autophagic vacuoles are not found in normal muscle fibers but are prominent in some muscle diseases where there is a deficiency of lysosomal enzymes as in acid maltase deficiency and Danon disease [30], impaired degradation of lysosomal contents due to an increase in lysosomal pH as in chloroquine myopathy [31], or when there is inhibition of phospholipid catabolism as occurs in vincristine myopathy [32]. Phosphatidylinositol 3-phosphate and phosphatidylinositol 3,5-bisphosphate, the substrates of myotubularins, are known to play important roles in vesicular trafficking and autophagy, and in recent years several myotubularin related proteins have been implicated as negative regulators of autophagy [33-37]. Although prominent upregulation of markers of autophagy has not yet been described as a general feature of XLMTM in humans, recent studies on two myotubularin-deficient mouse models have revealed significant abnormalities of the autophagy-lysosomal pathway [38,39]. Fetalvero *et al.* identified a profound increase of ubiquitin aggregates, LC3 levels, and activation of mTORC1 signaling associated with defective autophagy. Al-Qusairi *et al.* have shown the autophagic pathway is hyperactivated, however, late stages involving fusion of autophagosomes to lysosomes are blocked, leading to abnormal accumulations of autophagosomes similar to those seen in the affected Rottweiler’s muscles. Whether the particularly prominent accumulations in these dogs represents a general phenomenon, or perhaps reflects the effects of a genetic variant affecting some autophagy-related protein or other breed-specific finding, remains to be determined.

The clinical presentation of young dogs with either of the autosomal forms of CNM or of XLMTM is myopathic in nature (generalized weakness, muscle atrophy, short-strided gait) but these general signs of muscle disease are largely non-specific and typically cannot be used to diagnose the specific condition. If the serum CK activity is markedly elevated, a dystrophy rather than a congenital myopathy would be most likely. In either case, interpretation of muscle biopsies processed at a laboratory experienced in the diagnosis of neuromuscular diseases is of utmost importance as the congenital myopathies are defined by the presence of specific anatomic changes in frozen muscle biopsy sections [19]. Identification of an excessive variability in myofiber size, numerous myofibers containing centrally located nuclei resembling myotubes and the identification of ‘necklace fibers’ in the absence of dystrophic changes should alert the muscle pathologist to the diagnosis of either autosomal CNM or XLMTM. Then, differences in the ages of onset and rates of progression may provide clinical clues to allow differentiation of XLMTM from the known autosomal CNMs and may be used to guide the prioritization of genetic testing. XLMTM is restricted to males, and affected puppies present with a severe progressive weakness typically beginning in the first 2 months of life and requiring euthanasia by 4 to 5 months of age. In contrast, both Labrador retrievers with the *PTPLA* mutation [13] and Great Danes with *BIN1* mutation [14] have similar clinical phenotypes but tend to present at older ages and often survive well past 1 year of age [40,41]. Thus, the diagnosis cannot be made by physical examination alone but requires combined clinical and histopathological evaluations leading to selection of the proper genetic test to confirm the etiology.

The *MTM1* p.(Q384P) mutation appears to be restricted to this one kindred of Australian Rottweilers. Enquiries as to the history of any similar abnormalities in the dam’s and sire’s lines have not revealed any additional cases of congenital myopathy. Extensive pedigrees reveal ancestors including US, UK, German, and Swedish bred dogs. The grand dam on the dam side of the affected litters (I-3) was imported from Germany and has not produced any puppies with known abnormalities. DNA testing of the dam (II-2) of the two affected litters revealed that she was a heterozygous carrier for the *MTM1* p.(Q384P) mutation, yet testing of her dam’s (I-3) DNA demonstrated that she was not a carrier for the mutation. Given that the dam’s sire (I-2) was unaffected and able to breed, it is most likely that the *MTM1* mutation arose as a *de novo* event, either in the germ line of either grandparent I-2 or I-3, or in the dam II-2 during her embryonic development. Of the litter siblings of affected dogs, the males available for follow-up have not shown any abnormalities. The female littermates have either been neutered or not produced any known offspring. Nevertheless, any remaining unneutered female littermates in the affected litters are at 50% risk to carry the mutation and should either be neutered, or at least tested for the mutation. Regardless, unlike the situation with regard to the autosomal recessive *PTPLA* mutation that is distributed widely among geographically separated Labrador retrievers [42], it is highly unlikely that this particular *MTM1* mutation would be found in other Rottweilers, apart from direct lineal descendants of this single kindred.

## Conclusions

A progressive, non-dystrophic myopathy in male puppies from two litters of Rottweilers born in Australia represents the second genetically confirmed instance of XLMTM in dogs. The pathogenic mutation, *MTM1* p.(Q384P), lies three amino acids distal to the P-loop at the phosphatase active site and likely alters its structure through disruption of an adjacent alpha helix. Affected puppies have a similar clinical presentation and progressive course as previously reported Labrador retrievers with *MTM1* p.(N155K), suggesting that both models are representative of a variety of *MTM1* mutations as seen in human patients. Possible or probable XLMTM in dogs can be predicted for litters containing exclusively affected males with a skeletal myopathy of early onset and rapidly fatal course, together with characteristic skeletal muscle pathological findings, and should lead to diagnostic sequencing of the entire *MTM1* gene as this condition may be due to multiple independent mutational events regardless of breed or geographic origin.

## Abbreviations

CK: Creatine kinase
CNM: Centronuclear myopathy
COX: Cytochrome C oxidase
DHPR: Dihydropyridine receptor
PCR: Polymerase chain reaction
RyR1: Ryanodine receptor 1
SDH: Succinate dehydrogenase
SR: Sarcoplasmic reticulum
XLMTM: X-linked myotubular myopathy
